# “They accept me, because I was one of them”: formative qualitative research supporting the feasibility of peer-led outreach for people who use drugs in Dakar, Senegal

**DOI:** 10.1186/s12954-018-0214-1

**Published:** 2018-02-27

**Authors:** Camille May Stengel, Famara Mane, Andrew Guise, Magath Pouye, Monika Sigrist, Tim Rhodes

**Affiliations:** 1 University of Greenwich, Old Royal Naval College, Park Row, London, SE10 9LS UK; 2 Alliance Nationale des Communautés pour la Santé, Cité Keur Gorgui Villa 41, 10297 Dakar, Senegal; 3 Addison House, Guy’s Hospital, King’s College London, London, SE1 9RT UK; 4 International HIV/AIDS Alliance, Preece House, 91-101 Davigdor Rd, Brighton, BN3 1RE UK; 5 London School of Hygiene and Tropical Medicine, 15-17 Tavistock Place, London, WC1H 9SH UK

**Keywords:** Peer outreach, People who inject drugs, Peer-driven intervention, Peer educator, Substance use, West Africa

## Abstract

**Background:**

Peer outreach harm reduction initiatives are being developed with and for people who use drugs in Dakar, Senegal. This is in response to growing injecting drug use across the West Africa region and linked emerging epidemics of HIV and hepatitis C. We undertook formative qualitative research to explore the feasibility and potential of peer outreach in this context and in particular how outreach could be linked to fostering community-level processes of change.

**Methods:**

We undertook a total of 44 semi-structured qualitative interviews. Thirty-four interviews were with people who used drugs (comprised of 25 participants who had injected at least once in their life) and included 11 peer educators who delivered “awareness-raising” harm reduction activities. We also interviewed 10 service providers involved in the planning and monitoring of peer outreach initiatives. We used thematic analysis to identify key characteristics of how peer-led outreach is being delivered, beneficiary need, and the nature of the social networks in which the awareness-raising activities operate.

**Results:**

Through interviews with peer educators, people who use drugs, and service providers, four main overlapping themes are identified as follows: peer educators as a bridge to responsibilization through awareness-raising activities, awareness-raising activities as an enactment of recovery, awareness raising through social network diffusion, and the contexts and constraints of peer outreach engagement through awareness-raising activities.

**Conclusions:**

The study results suggest that peer education is on a trajectory to develop into a central role for harm reduction interventions in Dakar, Senegal. This research shows how peer education is bound in processes of responsibilization and self-change, which link to varying possibilities for risk reduction or recovery. For peer education to achieve a range of significant goals, broader structural and system changes should be implemented in the region. We caution that without such changes, awareness-raising activities and the role of peer educators may instead become part of state- and agency-sponsored processes of seeking to responsibilize individuals for health and harm reduction.

## Background

Drug use is emerging as a public health challenge across West Africa, including the spread of HIV through intravenous drug use [1–8]. In Senegal, HIV prevalence among people who inject drugs is estimated at 5.2% and hepatitis C (HCV) at 23%, respectively [3]. Women who use drugs (both injectors and non-injectors) are estimated to have 13% HIV prevalence and 32% HCV prevalence, markedly higher than their male counterparts (at 3% HIV prevalence and 23% HCV prevalence) [5]. Such high percentages suggest the need for effective responses to address the burden of blood-borne viruses among these subpopulations.

With injecting drug use documented in 28 African countries, and with increasing evidence of linked HIV epidemics in the Sub-Saharan region [9–14], there is a shift towards the incorporation of harm reduction policies [15, 16]. Harm reduction services such as needle and syringe exchange have been introduced in Senegal in the last few years. In the capital Dakar, the first state-financed opioid substitution therapy (OST) program in West Africa opened in 2014 [17]. In 2016, 110 people enrolled in the OST program [3]. Such harm reduction interventions are linked to outreach efforts designed to foster awareness and facilitate access to care services, including through peer-based outreach initiatives.

Global evidence supports the development and scale-up of combination interventions in HIV prevention and treatment for key populations of people who use and inject drugs, of which outreach is a key delivery mechanism [18–21]. Outreach seeks to provide health services to vulnerable or hidden populations in the settings where they are located [22–24]. One aim of outreach is to encourage people to change what is seen as “risk behavior” associating with injecting drug use and adopt safer harm reduction strategies that aim to prevent the transmission of blood-borne viruses [25–27]. Outreach can also potentially offer a “bridge” of health and social care and support to affected communities and their needs [28–30]. Outreach can thus be envisaged as a *social intervention* within broader structural intervention efforts to create “enabling environments” for change [31, 32].

Peer-based outreach initiatives involve people with current or former experience of drug use in the design, delivery, and/or advocacy of outreach interventions [18, 24]. There is a growing body of evidence specifically in support of peer-based outreach approaches [33–38]. Evidence supports a variety of models of peer-based outreach in initiating and sustaining community-level risk reduction, including what are known as “indigenous leader” and “peer-driven-intervention” models and “community health workers” [39–41]. Peer-based intervention has the potential to promote a ripple of community-level change by encouraging diffusion of behavioral norms through peer social networks, with the aim of enabling people who use drugs and their peers to drive public health interventions [29, 42–44]. However, in practice, the engagement of peers in outreach may not necessarily equate with a pathway of change oriented to community-level change.

The concern is that peer outreach may unwittingly further responsibility for health upon affected individuals and communities themselves rather than also orientate towards institutional and structural level changes [29, 45]. This process, known as responsibilization, can place additional burden on those providing harm reduction initiatives such as outreach and may reach beyond the remit or capacity of the position and ignore the larger risk environment context [46]. In the absence of structural change, and in the presence of often hostile social and economic environments, peer outreach may have perverse effects if the structural conditions in which it is delivered ration its potential [47]. This underscores the importance of situating the dynamics and potential linked to emergent peer-based outreach and also for exploring how peer outreach is experienced by those engaged in such interventions [48].

Peer-led approaches are increasingly being promoted in low-income settings as part of developing newly emerging outreach initiatives as integral to the HIV response for people who inject drugs [23, 49]. Peer “mentoring” has been shown as a promising HIV/AIDS awareness strategy in Senegal, although not in the context of harm reduction responses for people who inject drugs [50]. As a response to this gap, we conducted formative qualitative research as part of an academic-community partnership to situate peer outreach potential in the local context of Dakar, Senegal. This partnership brought together *Alliance Nationale des Communautés pour la Santé* (ANCS) and the London School of Hygiene and Tropical Medicine (LSHTM) in collaboration with drug information and awareness center *l’Association pour la promotion du Centre de sensibilisation et d’information sur les drogues Jacques Chirac de Thiaroye* (APSCID) and a self-organized advocacy group of people who use drugs.

The focus of this research was the peer outreach interventions being developed through partnership with ANCS (a civil society organization focused on responding to HIV in Senegal) and APSCID. The intervention involved training the civil society organization workers and peer educators to reach people who use or inject drugs through engaged conversations about harm reduction, HIV testing, sexual health, legal issues related to drugs, and the building of capacity for change [51]. These engaged conversations in the field are conceived locally as “awareness-raising” activities, and the primary means through which peer educators outreach is envisaged to enact change is through such awareness-raising activities (see below). The aims of the research study were to assess the feasibility and potential of peer outreach in this context and in particular how awareness raising could be linked to fostering community-level processes of change.

## Methods

The study utilized qualitative methods in three different geographical sites in Dakar, Senegal. Semi-structured interviews with people who use drugs, peer outreach workers, and delivery stakeholders were conducted with the aim to develop an in-depth understanding of contexts and experiences of peer outreach. Data were generated across three districts where drug users’ groups and/or APSCIDs trained peer outreach workers to actively work in Dakar.

### Data generation

Forty-four semi-structured interviews were conducted in July 2016. Please see Table 1 for more details of the participant categories. Some participants identified with more than one category. A total of 34 people who use drugs were interviewed: people who injected, smoked, and/or snorted the drugs heroin and/or cocaine (powder or crack), some in combination. Twenty-one people were enrolled in the methadone program. Two people stated they were HIV positive, and two other people disclosed that they had HCV. Eleven peer educators were also interviewed, 9 of whom identified as currently or formally using drugs. The average age of peer educators was 54 years old, and 9 of the 11 peer educators were men. In addition, the research team interviewed 10 service providers who were involved in the planning and monitoring of peer outreach. Two service providers also identified as peer educators.

**Table 1:** Participant categories. Total interviews = 44*

	People who use drugs	Peer educators	Service providers
Total	34	11	10
Of which			
Are women	12	2	3
Currently inject drugs	19	3	0
Enrolled in methadone	21	8	0
Are HIV positive	2	0	0
Are HCV positive	2	1	0

*Some participants are identified in more than one of these categories. They are included in all the categories that participants identified with

Interviews were conducted using a guide structured around key domains of interest for the experience of drug use and outreach: drugs used, transitions in to and through modes of use of drugs, family and community context, health status, service access, and interactions between peer outreach workers and beneficiaries. Interviews were conducted in either French or Wolof by trained research assistants and interviewers.

### Data analysis

Data were transcribed from oral Wolof to written French, and then translated to English. As a team, we pursued thematic coding and analysis of the transcripts in French and English. Data was categorized according to broad a priori domains of interest, developed through a review of literature on peer outreach in harm reduction as well as codes inductively derived from initial reading of the data. Within these key categories, we further coded and then elaborated themes that linked codes across the data [52, 53].

### Ethics

The study received ethical approval from the Ministry of Health in Senegal and LSHTM. All participants provided informed consent and received 2500 CFA West African Francs (equivalent to about 4.29 USD) in acknowledgement of their time and to cover transportation costs. All names used in this article are pseudonyms.

## Results

**Fig. 1:**
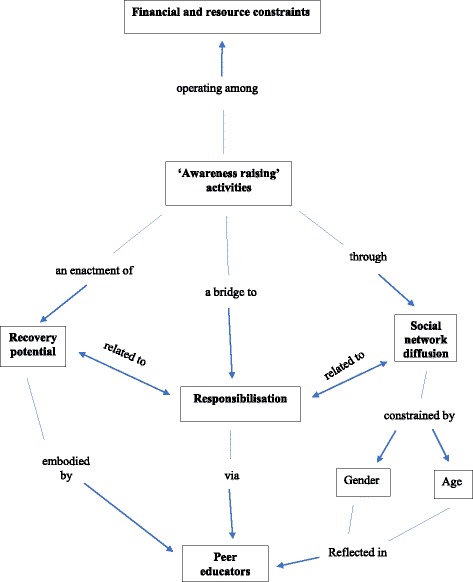
Conceptual map of findings

The feasibility and potential of peer outreach is explored in the findings through four overlapping themes: peer educators as a bridge to responsibilization through awareness-raising activities, awareness-raising activities as an enactment of recovery, awareness raising through social network diffusion, and the contexts and constraints of peer outreach engagement through awareness-raising activities. The relationship between these themes is illustrated in a conceptual map, outlined in Fig. 1 [54].

### Peer educators as a bridge to responsibilization through awareness-raising activities

As summarized in Table 2, the key attributes of the role of a peer as voiced by peers were constituted as a combination of experiential knowledge, being socially connected, being trusted, being a leader, and having responsibility.

**Table 2:** Peer educator attributes

Attributes of a peer educator	Participant quotations
Leadership	“troop leader” (Birame, peer educator) “capacity as an ambassador” (Malik, service provider)
Socially networked	“peer educators can quickly mobilize their peers” (Jakob, service provider) “keep a loyal audience to sensitize” (Sakou, peer educator)
Trustful	“people have trust for peers” (Musa, peer educator) “open, modest, trustful, understanding and tolerant” (Abacar, currently injects drugs and is a peer education beneficiary)
Tolerant	“tolerance, patience, doesn’t get upset or discouraged quickly, a good heart and helpful” (Saul, peer educator) “nice, tolerant, understanding, capable of helping others” (Fatou, a woman who uses drugs and a peer education beneficiary)
Knowledgeable	“teacher” (Babacar, currently injects drugs and is a peer education beneficiary) “educate others” (Ibrahim, peer educator)
Responsible	“because I tell myself I can’t save the world. I can bring my little stone, to be conscious of the dangers they [people who use drugs] are facing.” (Rose, peer educator) “tell other [drug users] what is good and what is bad” (Tito, currently uses drugs and a peer education beneficiary)

Experiential knowledge was cast as paramount, at once enabling a sense of understanding, shared and trusted connection, as well as legitimation in enacting or facilitating a responsibility to change:

Somebody who was with the fire and was burned in the fire with scars, is more capable to say to the others to ‘avoid the burn’ because he carries the marks. (Souleymane, peer educator)^1^

They accept me, because I was one of them: I was an injector, I injected myself, but now I’m taking methadone and we’re fighting the same battle. We have the same problems. (Amanita, peer educator)^2^

Souleymane recognizes that his experience leads to the responsibility for others not to make the same mistakes—his “marks” are scars that remind him and others of his experience. Likewise, Amanita sees herself as “fighting the same battle” as other people who use drugs, although she “was” *but no longer is* “one of them.” In pragmatic terms, such experiential knowledge and shared connection enables a bridge to communication and service provision, and many peer educators described themselves as being a “peer bridge.” Service providers too recognized this role:

We can’t integrate into this environment there [the drug using community]. We need a point of entrance. (Arame, local service provider)

Having the status of a peer bridge was presented as *legitimating* a role in the responsibilization of others in relation to their health and drug use. This included through demonstrating their own personal changes. Ablaye saw his peer role as showing promise to others by “preparing the way,” so that the outreach intervention—described and constituted locally as a process of awareness raising—can take place. He said that “it’s [the peer educators] who set the stage” for such awareness raising to occur through their presence as local peer leaders. In turn, Sakou and Ibrahim describe their experiential knowledge and the attributes this affords them almost as a form of currency to be exchanged. It is experience to be *used* as a means of responsibilizing others towards change, at the same time as enabling and reinforcing continued changes of the self for the better:

We can use the knowledge we have to sensitize them [people who use drugs] so that they stop or reduce the risks on the needle, their consumption. (Sakou, peer educator)

As the comment of one service provider also illustrates, peer education is at once a process of self and community responsibilization towards health. Arame sees peer educators as an opportunity to create

The links between HIV and drugs, [and] personal development – we [harm reduction services] have to show them [peer education beneficiaries] what they are, where they come from, what responsibilities they have in their lives. You really enhance personal development, self-esteem - all of which can really support an individual being, and [will be helpful] across their own responsibilities and their own commitments. (Arame, service provider)

For peer educator Birame, the benefits of awareness-raising activities are broader than health-related risk awareness and extend to the provision of social support. She says that peer educators “intervene where no one intervenes,” providing assistance directly not only in relation to drug use but also in relation to family, financial, and other everyday life concerns. The context and capacity for this assistance is discussed in the last theme of our findings.

Awareness raising primarily involved facilitated group discussions concerning risk awareness and the exchange of information about referrals. Awareness raising fell within the larger outreach activities that also involved home visits, counselling, needle and syringe exchanges, condom distribution, and support in relation to legal social and welfare issues. These activities were provided together based on need, rather than one-by-one isolation.

While awareness raising was framed as an initiative of “harm reduction,” peer educators were seen, and saw themselves, as key elements of a larger intervention that moved towards enabling abstinence from drug use. As described by Amar, peer education through awareness-raising activities was also envisaged as an indicator to promote addiction recovery and abstinence:

The peers [educators] who decide to help you, they can help you stop [using drugs] (Amar, who used to inject drugs).

We consider this theme of awareness raising by peer educators as an enactment of addiction recovery in the following subsection.

### Awareness-raising activities as an enactment of recovery

An emerging theme in the narration of peer education awareness raising as a site of responsibilization of self and of others is peer educator engagement as an act of addiction recovery potential. “Addiction recovery potential” is understood as manifestations of the process or journey to becoming a “recovered” person abstinent from drugs, such as engaging in awareness-raising activities, and are perceived to provide a source of hope to others currently using drugs or earlier along in their “journey.” Babacar, who injects drugs and is a peer educator beneficiary, envisions peer outreach as a means of helping people who are “sick” overcome their drug dependence. He describes people who use drugs—as he does himself—as “someone who is sick who must be helped” indicating that “they should also be given the methadone”.

Embodying self-recovery is the process to the enactment of addiction recovery potential in others, expressed through a disease model that views drug use as a “sickness.” Recovered peer educators exemplify a lifestyle change that is perceived as desirable and aspirational for others. Peer educator outreach engagement reproduces self-recovery efforts which capacitate others to realize or see their recovery potential. This is in large part what makes awareness-raising activities valuable and meaningful. As peer educator Musa explains, potentiating others is self-affirming:

What I like in this job is the fact of educating, of advising other users who are still using. I have been there, I had the experience. They saw that I’ve changed. I made them aware. I pray to God that they will change, as I did. (Musa, peer educator)

Peer outreach, then, can be a first step towards recovery, extending beyond awareness raising in relation to information-giving about risk reduction. Peer educators afforded this particular meaning given peer educators’ own experiences in having been sensitized to change through services involved in delivering the outreach intervention. Ibu, someone who used to inject drugs and is currently a peer educator, describes his sense of personal transformation through awareness raising affording his access to methadone treatment as follows:

I had a good situation, but as soon as I started using drugs, I found myself in a ridiculous situation. One day, a guy who was at [outreach delivery service], saw me so that I could quit using drugs, because I had lost a lot because of drugs. I lost my family, my friends, and my acquaintances. I went to the center, where I had training [as a peer educator]. Since then, I’ve changed, and I entered [methadone treatment]. Afterwards, I saw guys who were like me and who wanted to change, like they had seen that I changed, and they wanted to know where I had gone to be like that. I went to talk with them to make them know the path I used to get out [of using drugs]. (Ibu, peer educator)

Rather than a discourse of HIV prevention or harm reduction running parallel to an abstinence-oriented narrative of addiction recovery, peer outreach engagement operates locally as a site in which risk reduction fuses with recovery potential.

Awareness raising is specifically the *potential* of recovery, as some peer educators still use drugs, while others are on the methadone program and concurrently use stimulants like cocaine.

There are peers who use [drugs], it’s true. There are some peers who take methadone, they educate and have discussion groups, but afterwards they use with them [the beneficiaries], they will buy [cocaine] rocks. (Emmanuel, peer educator)

Three peer educators explicitly stated that they were currently using drugs, while others alluded to continued drug use. Framing peer education as focused around recovery potential, such continued use may appear to be in contradiction with the goals of awareness-raising activities. However, the symbolic role of peer educators allowed them to be perceived as conduits of responsibilization and recovery to their social networks, regardless of the individual educator’s actual drug use status.

### Awareness raising through social network diffusion

The behavioral change impact potential of peer outreach was envisaged as an effect of social network diffusion. Jakob, a provider of harm reduction services and coordinator of peer educator training, describes such change:

We have touched a lot of people who inject, which I believe also have touched other peers. I know if you touch the users on the ground and you talk to them about HIV awareness, awareness of hepatitis, how it is transmitted, how it should be avoided, where to go if infected, if you touch a set of drug users who participated in the [‘awareness raising’] activities, I believe they will be able to effectively share the information. (Jakob, service provider)

Peer educators’ awareness raising thus proceeded on the assumption that the awareness imparted will be shared within networked communities of people using drugs. At the same time, notions of “community” were multiple, both geographic and personal. Peer educator Souleymane, for example, described the personal benefits gained from his role as a peer educator being linked to his capacity to feel “useful, especially for my community.” Here, peer outreach via awareness raising is envisaged as impacting on the community as defined by individual peer educators. The diffusion potential of awareness-raising outreach is indexed to the personal network connections, particularly peer educators and the contacts they have with people who use drugs. Babacar, for instance, describes his network of diffusion potential in relation to the people he uses drugs around:

Usually it’s me only, sometimes with my buddies. Sometimes you come to buy and you find other people who had come to buy, and you stay because you know each other, since the smokers of [heroin] can know one another. We are one family; everyone knows the other. (Babacar, who injects drugs and is a peer educator beneficiary)

Similarly, Oumar describes his network as tightly bounded in relation to the house where he lived where groups of people used, bought, and sold drugs:

We were in [area in Dakar] in my elder brother; we can say that it was a smoking room. Only the Dieuki [‘junkies’] were grouped there together. They met just to smoke, or buy or sell. Anyone who came there was considered a smoker. Even if you do not smoke they will say that you are a smoker. We were at least five people constantly [ … ] Sometimes we are ten persons even if the dealer come we are five, fifteen, sometimes, twenty people in one room. (Oumar, who injects drugs and is peer education beneficiary)

Importantly, the peer educators linked to peer outreach awareness-raising efforts were primarily, though not exclusively, older men who were former or current heroin users that had transitioned from heron injecting to smoking. Women and young people who used drugs were largely absent from the networks contacted through peer outreach efforts. Ablaye, felt it harder for the peer outreach intervention to reach women, in part because women who use drugs are more socially hidden:

If we tallied up the people that we look after [through ‘awareness raising’], the women are in the minority, and yet there are female drug addicts who exist. Often, it’s the families who don’t accept them (Ablaye, peer educator)

Women who use drugs may be less connected to the injecting and smoking networks than their male counterparts, potentially limiting the reach awareness-raising activities through social network diffusion. Fatou, who currently smokes and injects drugs, explained, “I smoke in totally secrecy.” While she associated with a larger network of people who use drugs, who may “help each other out,” she accentuates that this larger network is not a community of “trust” and that her smoking of heroin and crack cocaine was always alone. The lack of networked connection to women who use drugs was corroborated by others. For instance, Kuta, a woman who currently injected drugs, had never heard of, or had any engagement with, the peer outreach intervention.

Similarly, it was acknowledged that the peer education intervention was not connecting up with younger people involved in drug use. This does not mean that young people using drugs do not exist locally, it is more the case that the peer outreach intervention had its connections with other networks of (older, male) people who use drugs, which made reaching young people who use drugs difficult. Service provider Malik, who provides employment skills training for former people who use drugs, identifies how it is challenging to recruit people under 40 years old:

The difficulty we have concerning this training, is that we have a target population that is not very young [ … ] at 25 years you are still very young, you want to enjoy yourself [ … ] but when you are 40 and 45 years old, you become aware and think it is time to stop [drugs]. (Malik, service provider)

In addition, those working as peer educators, especially those engaging in a process of recovery from addiction, may have become socially or materially distanced from those with whom they have networked connections. This could partially explain why most peer educators were over 50 years old and mainly men—they were simply not connected with different iterations and new social networks of drug use in Dakar. In this context, reaching more diverse populations of people who use drugs like young people and women were one of the challenges in awareness-raising activities.

### The contexts and constraints of peer outreach engagement through awareness-raising activities

Linked in large part to the underlying context of poverty for people who use drugs, the potential for financial assistance was an integral part of peer education through awareness-raising activities. Financial assistance was commonly provided on an informal basis by peer educators to beneficiaries of the outreach intervention. Peer educator Emmanuel saw providing financial assistance to beneficiaries outside of the talks as “part of the job.” He explained how

Only just today, a [person who uses drugs] stopped me because he wanted to use, but did not have enough money, so I gave it to him. That’s part of the job, too. There are peers who do it [give money], there are others who don’t. But when you help them, they thank you. When you don’t help them, they say nothing to you. Every time you listen to their conversations, you will know who helps them and who doesn’t. And they ask you for money all the time. (Emmanuel, peer educator)

Peer educators were officially offered cash reimbursements from the organizations running the awareness-raising activities. These reimbursements were designed to be sufficient to cover the costs of their transportation (between 2000 and 5000 CFA, around 3.40 to 8.50 USD).

Aside from transportation reimbursement, the peer educator role was ostensibly a voluntary position. The beneficiaries of peer outreach were aware of the voluntary nature of peer education. Service provider Malik described how peer educators were told during their awareness-raising delivery training

They are always told that the activities which we undertake concerning this program, if they manage to seek something *they should go with their own funds* (italics added).

The role of a peer educator was not a viable job opportunity, but an altruistic way to occupy a leadership position of sharing harm reduction information to their social networks. Fatou, who smokes and injects drugs, recognized that peer educators do not receive adequate compensation for their activities, asserting

Yes, [the peer educator] gives me some money. That’s why I say that they should be [financially] supported, too.

Material context is thus a key dynamic affecting engagement in peer outreach, both from the perspective of providers and receivers of the intervention.

Findings also point to peer educators having to navigate beneficiary needs and demands which extend beyond the capacities that the low-resource outreach services can provide. Peer educator Rose valued the supportive aspects of peer education work, but found that she could not fulfil the different roles that people who use drugs need. Here, she notes how the peer outreach intervention is limited in relation to the extent of medical expertise and services it can offer:

I always say when I give talks and run support groups: I am not a doctor; I am not a doctor. Sometimes they pull you aside to tell you all their problems. Or they just give you a prescription, and you see really embarrassing situations. You’re handcuffed, you can’t act, there’s no more support. That’s a big gap. (Rose, peer educator)

Other peer educators confirmed that they provided support directly to beneficiaries on a voluntary basis over and above that delivered formally through the outreach intervention. Service provider Youssou, for example, provided some medical care and prescriptions to beneficiaries who could not pay. This service extended beyond the boundaries of peer education, but was provided as a response to beneficiary’s needs. Such a finding suggests the need to support a widening of the peer education intervention beyond the current awareness-raising activities.

As noted above, the peer outreach awareness-raising sessions emphasized information giving and awareness raising as a means of facilitating risk reduction and behavior change. The provision of new needles and syringes were made available post-awareness-raising sessions for free from peer educators directly or via linked health care services, but distribution was limited based on variable availability. Service provider Djibo acknowledged how

In the past there was not a system of collecting syringes for being incinerated. The user injected themselves and threw the syringe unconsciously

However, he explained that through the awareness-raising talks, the service providers and peer educators introduced the idea of safely discarding and disposing of syringes. Service provider Arame identified “the distribution of syringes” as a main area of improvement for awareness-raising activities.

In addition to the need to scale-up needle and syringe distribution as an integrated part of outreach, peer educator accounts in this research and recent studies in Canada point to a need for harm reduction materials for smokers of crack cocaine [55]. Both Fatou and Modou described how they made their own pipes for smoking crack cocaine. Oumar, who smoked as well as injected, explained how in his network of people who used drugs

The pipe is often shared. They [the peer educators] often tell us that this is not hygienic to share the same pipe, that we each must have his [own] pipe.

As with HIV risk and prevention linked to needle and syringe sharing, the intervention’s change potential is indexed to its capacity to provide the material means for change.

Lastly, some accounts suggest that the overall aims and emphasis of the intervention was framed by the coordinating agencies rather than through the involvement of the individual peer educators themselves. While peer educators played a key role in delivering awareness raising, they felt themselves less responsible for creating the content that they shared with their imagined community of peers. Peer educator Emmanuel commented on the development of the topics and educational messages framing peer outreach awareness-raising sessions as “there’s nothing that we [peer educators] invent or create for ourselves.”

It was also noted that networks of people who use drugs locally were not explicitly included in the design of the field manuals used to guide the content of peer outreach intervention. Here then, the intervention potentially acts as a site of responsibilization of peer educators to communicate particular educational messages and change (drawing on narratives of self-empowerment and recovery as described above) but without fully enabling their involvement to mediate how the peer outreach awareness raising is designed and delivered.

## Discussion

The results of this study help facilitate understanding of the experiences and contexts for peer-based outreach for harm reduction in Dakar, Senegal. These results suggest particular opportunities for peer education to have community-wide effects in Dakar while also recognizing the potential for peer outreach to become a process of seeking to responsibilize vulnerable individuals for their health in the absence of broader supportive systems change.

A feature of peer-led educators in Dakar was described by peers and beneficiaries alike as involving particular trusting relationships, and moreover, that this trust is generated and grounded in valuing of shared experience [56, 57]. Peer educators occupy the unique position simultaneously transcending those in the community through the process of responsibilization. Through the medium of awareness-raising activities, and as a result of their (perceived or real) “reformed” risk behavior, peer educators both shared experiences but are also burdened with the responsibility of communicating behavior change to networks of people who used drugs, as well as presenting themselves as ambassadors of change on the road to recovery.

The role of a peer educator is also a process of modelling a non-stigmatized identity, and so demonstrating how drug use can form part of a process of being accepted within a community [30]. The role of bridge for peer outreach workers then refers not only to a metaphor for covering a previously impassible physical obstacle, but also in providing a route to which people using drugs can re-identify with a broader community [24]. The results from the study show how awareness-raising activities through peer educators goes beyond a linear process of diffusing through a social network, and instead fosters a broader de-stigmatization of people who use drugs and their meaningful involvement in interventions, thus creating conditions for community building [29, 31].

The context for peer education shaped the potential for achieving certain goals and reaching social networked groups of people who use drugs. Specifically, the orientation of awareness raising was aligned with messages of a bio-medical context of recovery and peer educators’ embodiment of a reformed person who used drugs [58, 59]. Such explicit emphasis on recovery creates opportunities and potential as it relates to some experiences of drug use, while limiting others. This could be linked to harm reduction and peer education being a nascent (yet growing) discourse within Senegal, [3, 5, 50] alongside and overlapping with efforts to support recovery and abstinence. The underlying focus of recovery and drug treatment may then limit the potential for peers to engage with people not aligned to these goals, and therefore limit the scope and reach of diverse harm reduction interventions [60, 61].

The prospect for diffusion of risk reductive norms through a social network is potentially limited by processes of social distance and identity that lead to limited network contact. Findings from this study reveal how young people and women were seen as having limited contact with the peer educators, who were predominantly older men. Physically and culturally apparent notions of identity and their effect may be exaggerated in their influence, when such differentiation becomes inevitable through other processes. Findings from this study suggest that peer educators should be diversified to include more diverse age and gender representation among people who use drugs, who might have different needs than those currently acting as peer educators [62, 63]. Given the higher rates of HIV among women who use drugs in Senegal in a previous study, future peer educator recruitment and outreach should be focused on a gender inclusive approach [5].

These discursive constructs come within material constraints. The specific limited availability of needles and syringes is hinting at the broader resource constraints in which supplies are constrained, not responding to varying needs such as smokers, and indicative of experiences of poverty within the larger risk environment [46]. Constraints also resulted in financial expectations not matching economic reality, suggesting the need for long-term sustainable funding for awareness-raising activities and to adequately support peer educators [64]. The responsibilization of people to change their own risk behaviors, while ignoring the lack of sustainable and far-reaching structural supports, perpetuates a risk environment among people who inject drugs in Senegal.

There are important limitations to this study. Potentially, a loss of nuance occurred in the data due to the translation between spoken Wolof, written French, and written English. Attempts to mitigate this included analysis of French transcripts by fluent Wolof speakers, transcribers’ cross-checking audio recordings when context was unclear, and an iterative feedback loop between English and French analysis team to clarify any potential inconsistencies. As the data from this qualitative research was part of a feasibility study, all findings should be interpreted with caution and not assume representation of all people who use and especially people who inject drugs in Senegal. The small sample size and degree of familiarity between some participants means other people’s voices were not heard. That being said, the interviews with people who inject drugs and health care providers present a salient depiction of the lives and needs of peer outreach in Dakar.

## Conclusions

Data from this qualitative research, the first of its kind in Senegal, reveals a composite picture of peer education outreach that is both community driven and an indirect function of responsibilization. If sustainable financing and collaboration with groups who represent people who use drugs continues, there is promising potential for peer education and awareness raising to be expanded and further developed into meaningful harm reduction practice for a diverse range of the population of people who use drugs. However, the findings also point to how peer educators may become the focus of expectations—for services, for resources—that are unable to be met in the absence of broader system and structural changes, themselves contingent on a massive increase on resource availability in Senegal.

## Abbreviations

ANCS: Alliance Nationale des Communautés pour la Santé
APSCID: l’Association pour la promotion du Centre de sensibilisation et d’information sur les drogues Jacques Chirac de Thiaroye
CFA: Communauté Financière Africaine (African Financial Community)
HCV: Hepatitis C virus
HIV: Human immunodeficiency virus
USD: United States dollar

## Footnotes

1 This quote is a metaphor alluding to a popular Wolof proverb, which roughly translated into English is “only he who tastes it can describe it.”

2 Participants in this study are referred to as the following classifications: person who used to inject drugs, person who injects drugs, person who used to use drugs, person who uses drugs, peer educator, and local service provider. These labels are not meant to be all encompassing or stigmatizing to the individual, but to categorize their main role(s) within this study in a streamlined manner. We have avoided the use of acronyms in an attempt to mitigate any stigmatization potentially associated with the categories.
